# Supplementary material for: A cyclin-dependent kinase-mediated phosphorylation switch of disordered protein condensation
Source: Nat Commun. 2023 Oct 9;14:6316. doi: 10.1038/s41467-023-42049-0 (PMC10562473; doi:10.1038/s41467-023-42049-0)
Supplement: Supplementary file 1 — Supplementary Information [file 41467_2023_42049_MOESM1_ESM.pdf]

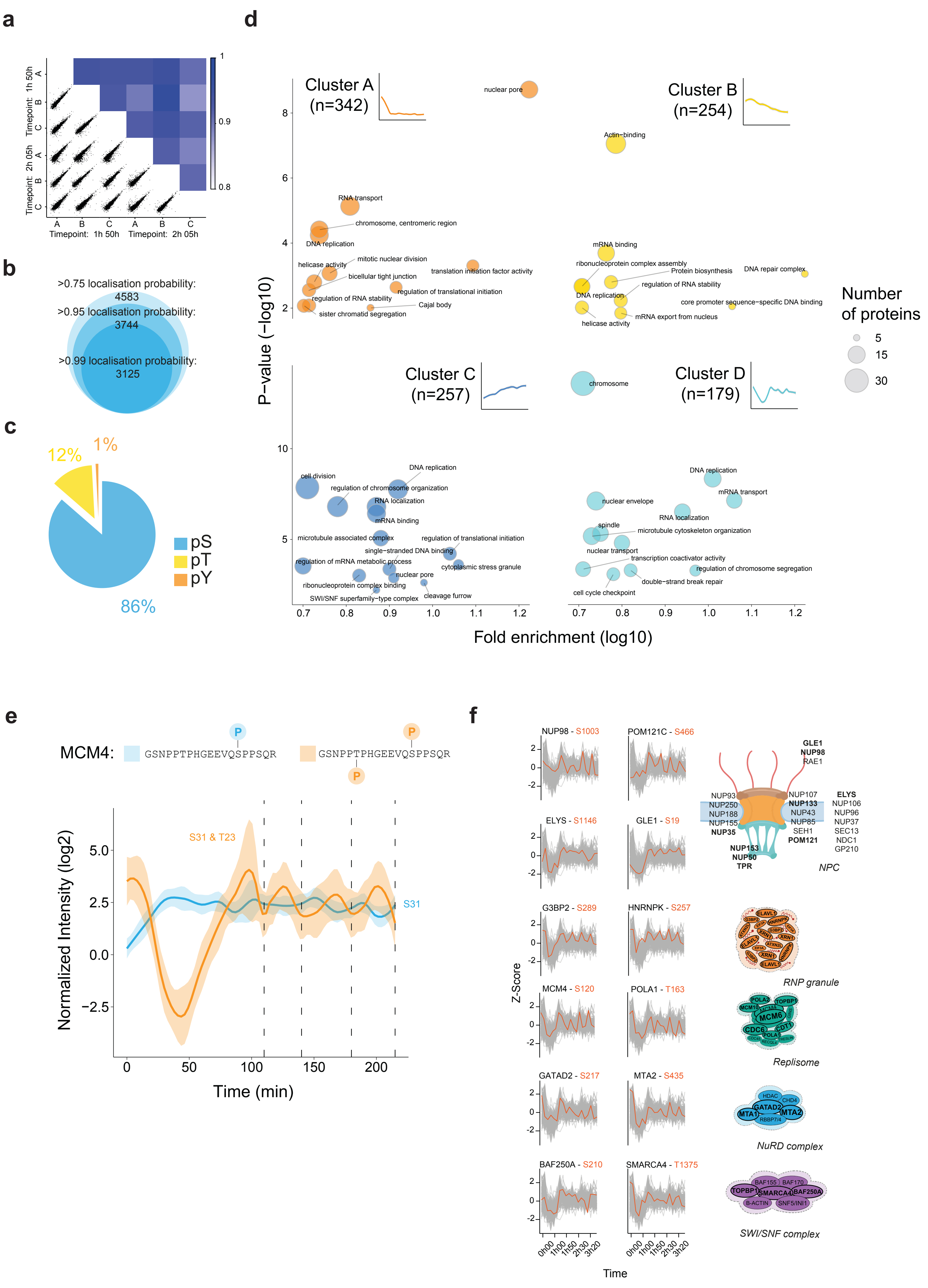
a
Timepoint: 1h 50h
Timepoint: 2h 05h
A
B
C
Timepoint: 1h 50h
Timepoint: 2h 05h
1
0.9
0.8
b
>0.75 localisation probability:
4583
>0.95 localisation probability:
3744
>0.99 localisation probability:
3125
c
12%
1%
86%
pS
pT
pY
d
Cluster A
(n=342)
Cluster B
(n=254)
Cluster C
(n=257)
Cluster D
(n=179)
nuclear pore
Actin−binding
RNA transport
chromosome, centromeric region
DNA replication
mitotic nuclear division
helicase activity
bicellular tight junction
translation initiation factor activity
regulation of translational initiation
regulation of RNA stability
Cajal body
sister chromatid segregation
mRNA binding
ribonucleoprotein complex assembly
Protein biosynthesis
DNA repair complex
regulation of RNA stability
core promoter sequence−specific DNA binding
mRNA export from nucleus
helicase activity
chromosome
cell division
regulation of chromosome organization
RNA localization
mRNA binding
microtubule associated complex
regulation of translational initiation
single−stranded DNA binding
regulation of mRNA metabolic process
cytoplasmic stress granule
nuclear pore
ribonucleoprotein complex binding
cleavage furrow
SWI/SNF superfamily−type complex
mRNA transport
nuclear envelope
spindle
microtubule cytoskeleton organization
nuclear transport
transcription coactivator activity
regulation of chromosome segregation
double−strand break repair
cell cycle checkpoint
P−value (−log10)
Fold enrichment (log10)
Number of proteins
5
15
30
e
MCM4:
GSNPPTPHGEEVQSPPSQR
GSNPPTPHGEEVQSPPSQR
P
S31 & T23
S31
Normalized Intensity (log2)
Time (min)
f
NUP98 - S1003
POM121C - S466
ELYS - S1146
GLE1 - S19
G3BP2 - S289
HNRNPK - S257
MCM4 - S120
POLA1 - T163
GATAD2 - S217
MTA2 - S435
BAF250A - S210
SMARCA4 - T1375
Z-Score
Time
0h00
1h00
1h50
2h30
3h20
GLE1
NUP98
RAE1
NUP93
NUP250
NUP188
NUP155
NUP35
NUP107
NUP133
NUP43
NUP85
SEH1
POM121
ELYS
NUP106
NUP96
NUP37
SEC13
NDC1
GP210
NUP153
NUP50
TPR
NPC
RNP granule
Replisome
NuRD complex
SWI/SNF complex

Supplementary Fig. 1. Phosphosite dynamics correlates with cell cycle phases. a. Correlation coefficients for two randomly selected time points. **b.** Total number of phosphosites detected and their distribution according to the site localisation probability score. **c.** Distribution of phosphosites identified among serine, threonine and tyrosine residues. **d.** Scatter plots of significantly enriched (Fisher's exact test with Bonferroni correction, $p<0.05$) GO (BP, MF, CC, Uniprot keywords) terms for all dynamic phosphosites per cluster in the in vivo experiment, presenting the fold-enrichment of specific terms vs statistical significance. The size of the circles correlates with the number of proteins associated with the specific term. More details and the full list of enriched GO terms per cluster is found in Supplementary Dataset 1. **e.** *In vivo* reciprocal trends of singly- and multi-phosphorylated peptides carrying phosphorylated T23 and S31 of MCM4 (dashed lines depict the time points of cell division): orange curves, the trend of T23 and S31 in the multi-phosphorylated peptide; blue curve, the trend of S31 in the singly-phosphorylated peptide. **f.** Examples of proteins with known association showing similar oscillating phosphorylation. Plots highlight the dynamic trend of the cluster (grey) and selected phosphosites (orange) over time. Right, illustrations of protein complexes formed by the proteins undergoing dynamic phosphorylation. Proteins highlighted in bold show at least one oscillating phosphosite in our dataset. Source data are provided as a Source Data file.

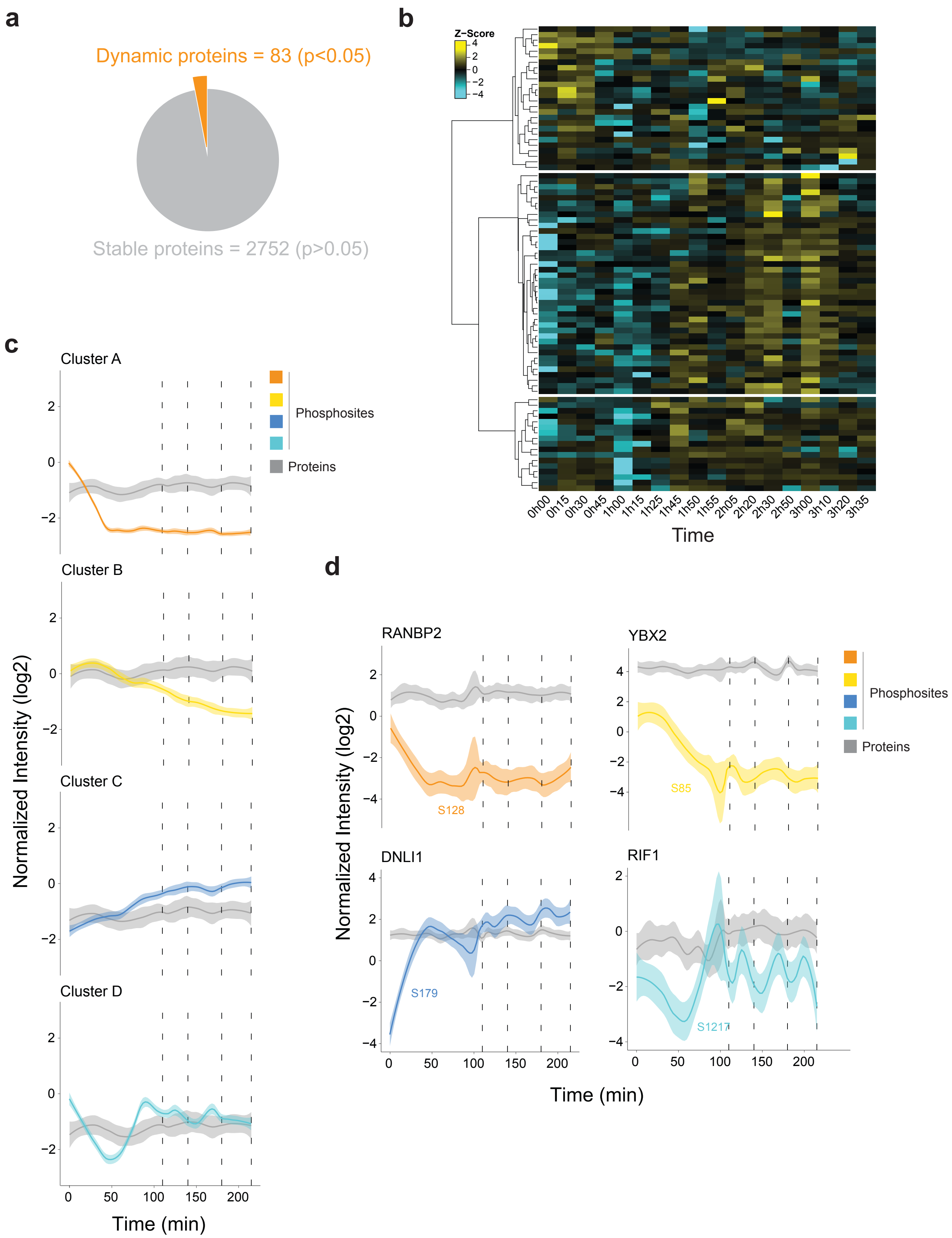

Supplementary Fig. 2. Variation in the total proteome versus the phosphoproteome in early embryonic cell cycles. a. Total proteome analysis reveals 83 proteins out of 2835 showing significant changes in abundance (ANOVA, Benjamini-Hochberg correction, FDR 0.05) over the time course. **b.** Heat map showing abundance of the variable proteins over the time course. **c.** Comparison of dynamic variations in total protein compared to total phosphosites from the four clusters shown in Figure 1c (dashed lines depict the time points of cell division). **d.** Examples of dynamics of individual phosphosites from the four clusters shown in Figure 1c and levels of the corresponding protein (dashed lines depict the time points of cell division). Source data are provided as a Source Data file.

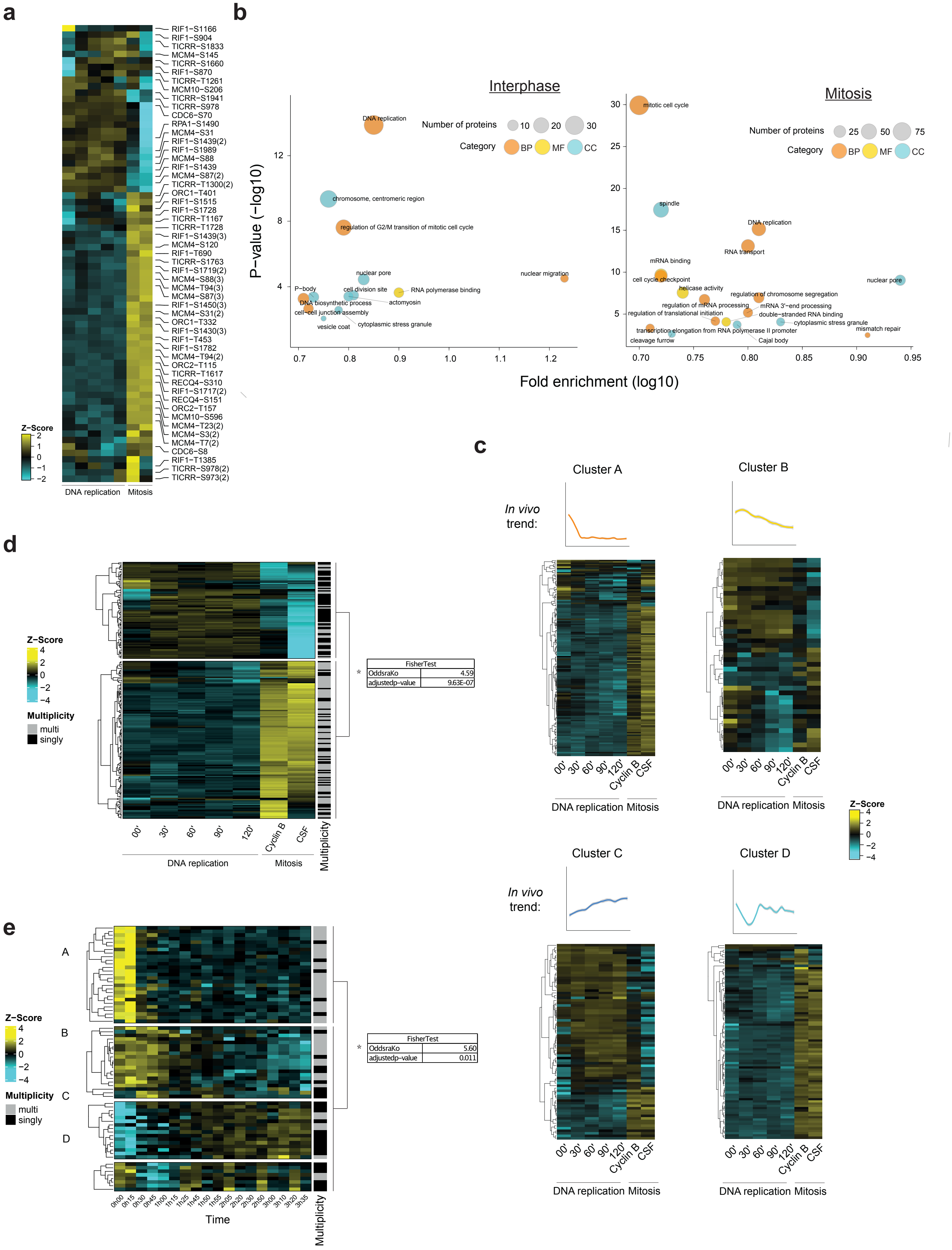
a
RIF1−S1166
RIF1−S904
TICRR−S1833
MCM4−S145
TICRR−S1660
RIF1−S870
TICRR−T1261
MCM10−S206
TICRR−S1941
TICRR−S978
CDC6−S70
RPA1−S1490
MCM4−S31
RIF1−S1439(2)
RIF1−S1989
MCM4−S88
RIF1−S1439
MCM4−S87(2)
TICRR−T1300(2)
ORC1−T401
RIF1−S1515
RIF1−S1728
TICRR−T1167
TICRR−T1728
RIF1−S1439(3)
MCM4−S120
RIF1−T690
TICRR−S1763
RIF1−S1719(2)
MCM4−S88(3)
MCM4−T94(3)
MCM4−S87(3)
RIF1−S1450(3)
MCM4−S31(2)
ORC1−T332
RIF1−S1430(3)
RIF1−T453
RIF1−S1782
MCM4−T94(2)
ORC2−T115
TICRR−T1617
RECQ4−S310
RIF1−S1717(2)
RECQ4−S151
ORC2−T157
MCM10−S596
MCM4−T23(2)
MCM4−S3(2)
MCM4−T7(2)
CDC6−S8
RIF1−T1385
TICRR−S978(2)
TICRR−S973(2)
Z−Score
2
1
0
−1
−2
DNA replication
Mitosis
b
Interphase
Number of proteins
10
20
30
Category
BP
MF
CC
DNA replication
chromosome, centromeric region
regulation of G2/M transition of mitotic cell cycle
nuclear pore
nuclear migration
P−body
cell division site
RNA polymerase binding
DNA biosynthetic process
actomyosin
cell−cell junction assembly
vesicle coat
cytoplasmic stress granule
P−value (−log10)
Mitosis
Number of proteins
25
50
75
Category
BP
MF
CC
mitotic cell cycle
spindle
DNA replication
RNA transport
mRNA binding
cell cycle checkpoint
nuclear pore
helicase activity
regulation of chromosome segregation
regulation of mRNA processing
mRNA 3'−end processing
regulation of translational initiation
double−stranded RNA binding
cytoplasmic stress granule
transcription elongation from RNA polymerase II promoter
mismatch repair
cleavage furrow
Cajal body
Fold enrichment (log10)
c
Cluster A
Cluster B
In vivo trend:
00' 30' 60' 90' 120' Cyclin B CSF
DNA replication
Mitosis
Z−Score
4
2
0
−2
−4
Cluster C
Cluster D
In vivo trend:
d
Z−Score
4
2
0
−2
−4
Multiplicity
multi
singly
FisherTest
OddsraKo 4.59
adjustedp−value 9.63E−07
*
00' 30' 60' 90' 120' Cyclin B CSF
DNA replication
Mitosis
Multiplicity
e
A
B
C
D
Z−Score
4
2
0
−2
−4
Multiplicity
multi
singly
FisherTest
OddsraKo 5.60
adjustedp−value 0.011
*
0h00 0h15 0h30 0h45 1h00 1h15 1h25 1h45 1h50 1h55 2h05 2h20 2h30 2h50 3h00 3h10 3h20 3h35
Time
Multiplicity

Supplementary Fig. 3. *In vitro* phosphoproteomics discriminates interphase and mitotic phosphorylation. **a.** Heatmap of dynamic phosphosites detected in DNA replication factors. **b.** Scatter plots of significantly enriched (Fisher's exact test with Bonferroni correction, $p<0.05$) GO terms for all dynamic phosphosites upregulated during interphase (top) and mitosis (bottom), presented as fold-enrichment of specific terms vs statistical significance. The size of the circles correlates with the number of proteins associated with the specific term, while the color corresponds to the GO term category. **c.** Behaviour of *in vivo* dynamic phosphosites (top) in *in vitro* experiments. **d.** Subset of phosphorylation sites detected in both singly and multi-phosphorylated peptides *in vitro*, showing enrichment of multi-phosphorylated species in mitotic samples. The common Odds Ratio was calculated with the Fisher's test and p-values were adjusted using the Benjamini-Hochberg method. **e**. As (d) but for *in vivo* data, showing the enrichment of phosphorylation sites from multi-phosphorylated peptides in cluster A (related to meiotic metaphase II). Source data are provided as a Source Data file.

a

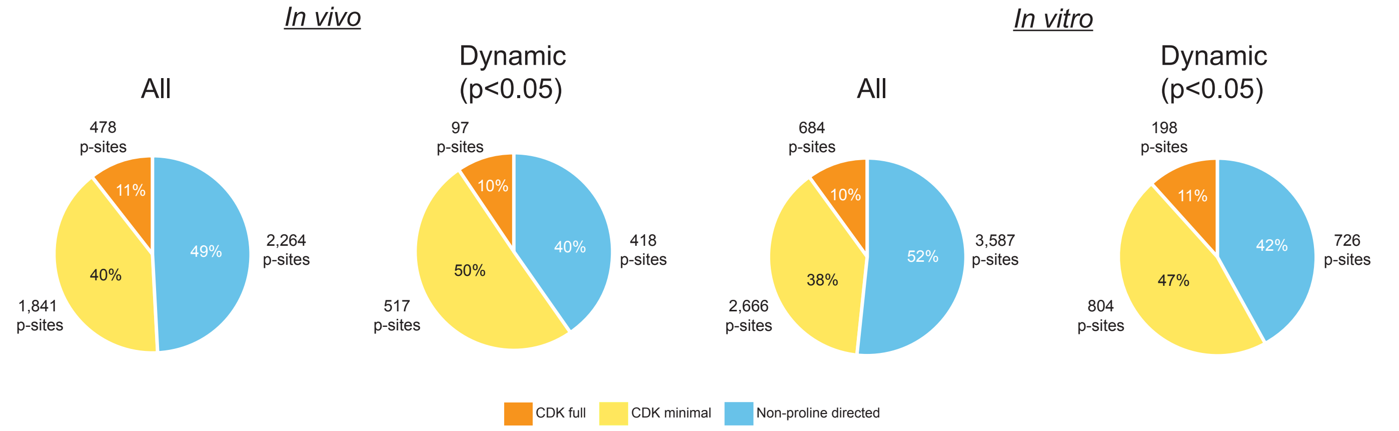

b

In vivo

	PLK	AURKA/B	CK1/2	DDK	CDK full	CDK min
Cluster A (n=342)	14	12	97	12	14	205
Cluster B (n=254)	11	27	67	12	16	124
Cluster C (n=257)	9	29	72	31	36	135
Cluster D (n=179)	7	5	51	4	31	150
Total (n=1032)	41	73	287	59	97	614

In vitro

		PLK	AURKA/B	CK1/2	DDK	CDK full	CDK min
M-phase	Cluster 1 (n=161)	23	7	56	5	12	67
	Cluster 2 (n=172)	5	6	62	6	23	114
	Cluster 3 (n=702)	16	23	224	5	123	565
Interphase	Cluster 4 (n=151)	13	52	48	8	6	20
	Cluster 5 (n=433)	15	34	136	67	25	196
	Cluster 6 (n=109)	6	9	36	14	9	40
	Total (n=1728)	78	131	562	105	198	1002

c

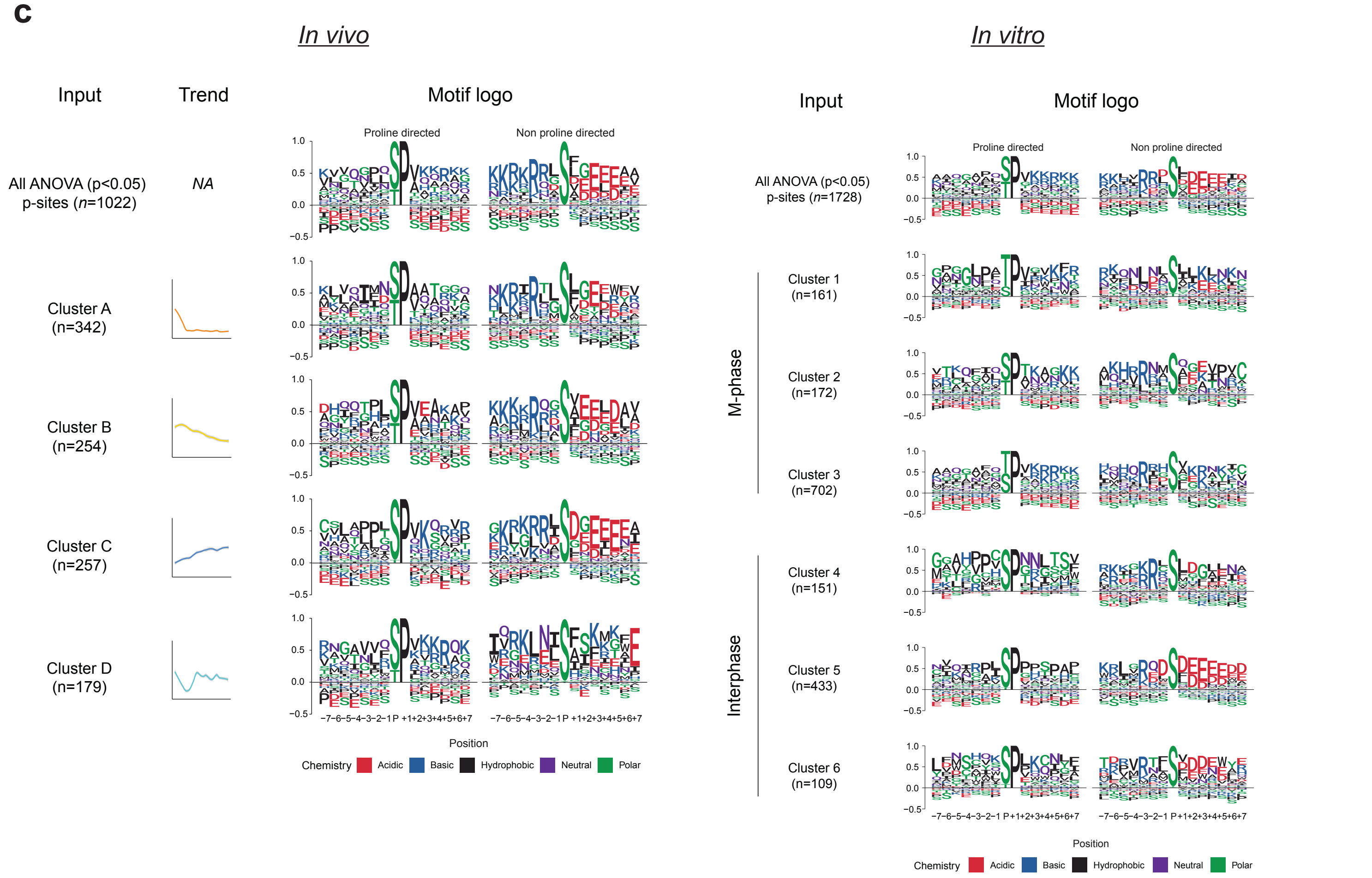

d

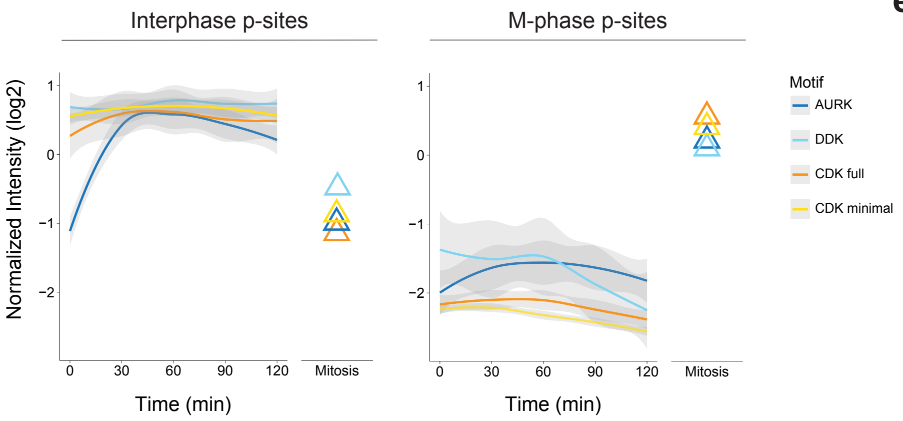

e

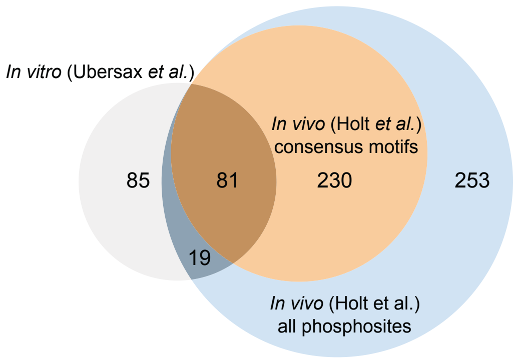

Supplementary Fig. 4. CDK consensus phosphosites dominate the early embryo phosphoproteome; a. Distribution of potential CDK targets among all detected phosphosites and dynamic phosphosites, *in vivo* (embryo, left) and *in vitro* (egg extract, right). **b.** Observed phosphorylation motifs in the dynamic phosphoproteome *in vivo* (left) and *in vitro* (right). See methods for details. Note: in some cases, the sum of consensus sites exceeds the number of phosphosites due to redundancy between motif predictions. **c.** Sequence motif logo for all dynamic phosphosites and for each of the clusters shown in Figure 1c and 1e, for the *in vivo* and *in vitro* experiments, respectively. Motifs are shown separately for proline-directed and non-proline-directed phosphosites. **d.** Dynamic trend of phosphorylations of potential kinase targets in egg extract S-phase clusters (4-6, left) and in mitotic clusters (1-3, right). **e.** Venn diagram of observed *in vitro* (grey) and *in vivo* (blue) yeast CDK targets. *In vivo* targets showing CDK minimal consensus motif phosphorylations are highlighted in orange. Source data are provided as a Source Data file.

a

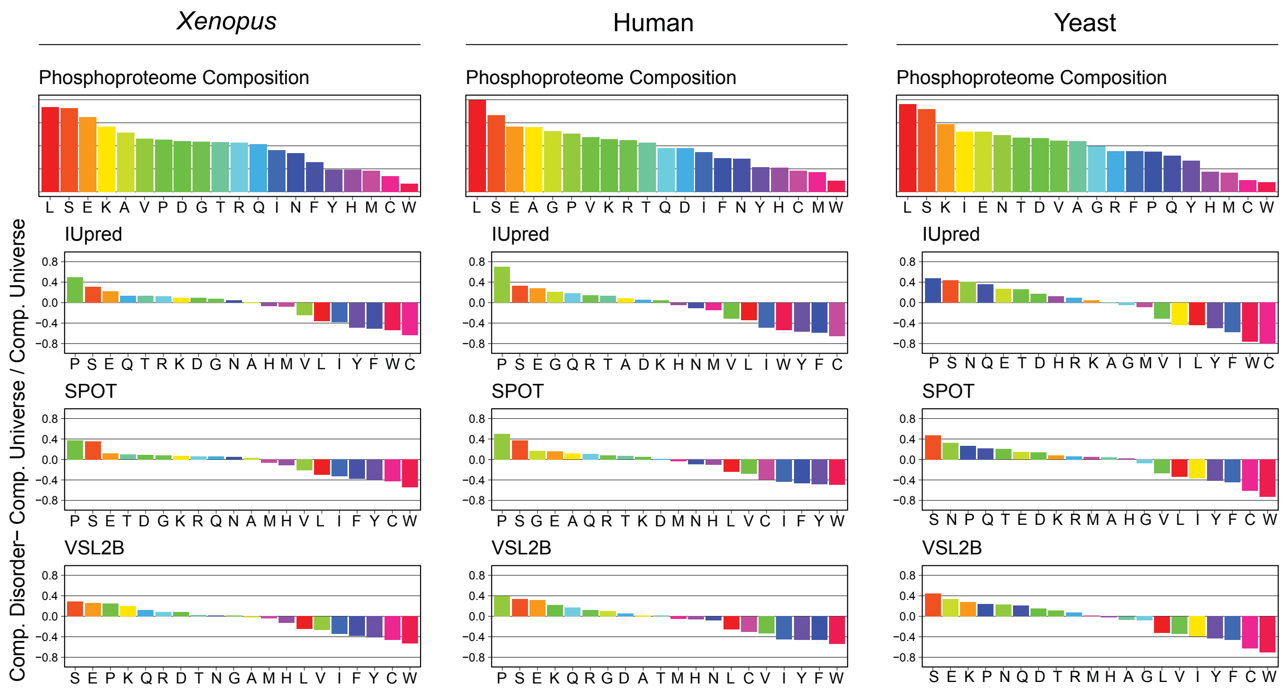

b

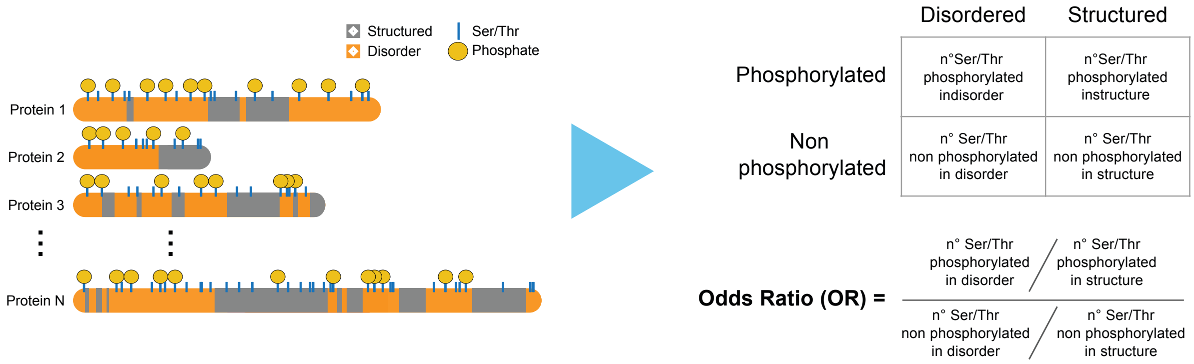

c

	IDR predictor	Disorder fraction	P-value	Odds ratio
Xenopus dynamic	IUPred	0.234	6.6E-51	2.70
	SPOT	0.277	1.3E-83	3.86
	VSL2b	0.381	2.2E-78	4.38
Human CDK targets	IUPred	0.259	4.9E-36	2.18
	SPOT	0.326	3.2E-56	3.05
	VSL2b	0.454	2.6E-54	3.84
Yeast CDK targets	IUPred	0.200	2.6E-31	4.89
	SPOT	0.277	7.8E-45	25.14
	VSL2b	0.380	8.6E-36	51.95

	IDR predictor	Disorder fraction	P-value	Odds ratio
Human MAPK targets	IUPred	0.259	6.2E-50	2.92
	SPOT	0.326	1.6E-45	2.91
	VSL2b	0.454	1.1E-45	3.57
Human AURK targets	IUPred	0.259	7.7E-15	1.77
	SPOT	0.326	6.0E-40	3.10
	VSL2b	0.454	1.6E-20	2.41
Human PLK targets	IUPred	0.259	1.0E-19	1.78
	SPOT	0.326	2.3E-51	2.98
	VSL2b	0.454	7.1E-42	3.14
Human NEK targets	IUPred	0.259	5.0E-01	0.84
	SPOT	0.326	7.4E-01	1.10
	VSL2b	0.454	3.5E-01	0.80
Human DYRK targets	IUPred	0.259	3.7E-04	2.43
	SPOT	0.326	3.2E-04	2.54
	VSL2b	0.454	6.2E-05	4.01

d

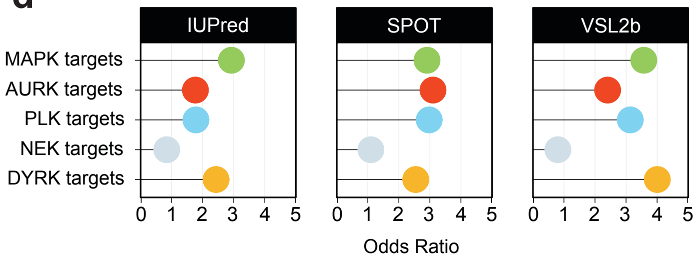

e

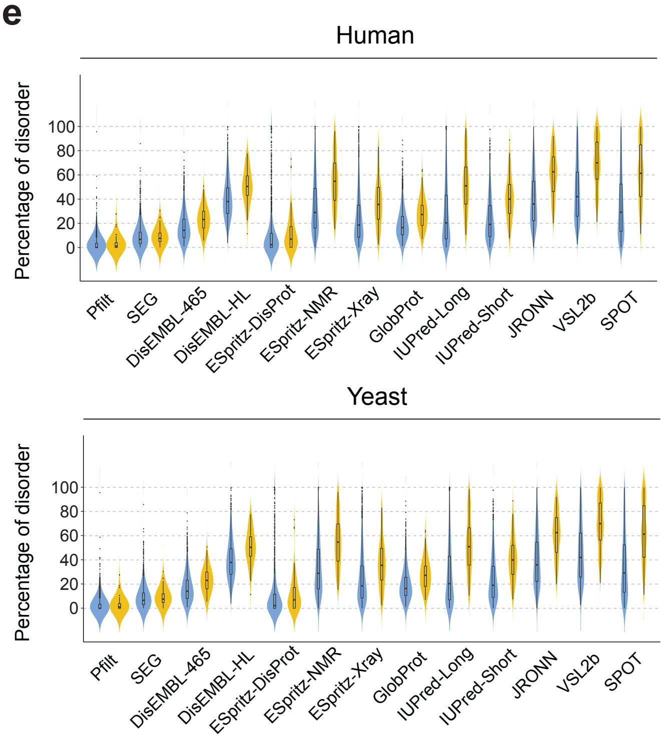

Supplementary Fig. 5. Cell cycle phosphorylation occurs preferentially in IDRs. a. Differential amino acid composition (see methods) in disordered regions for *Xenopus*, human and yeast determined with three IDR predictors. Amino acids are coloured in a rainbow pattern according to their relative abundance in each phosphoproteome. Disruptions of the rainbow pattern show specific compositional signatures for IDRs. **b.** Scheme of the Odds Ratio analysis using contingency tables. The counts of phosphorylated Ser/Thr for all the proteins for each set (CDK-mediated in yeast/human, or dynamic in *Xenopus*, and other cell cycle-related kinases in human), in disordered and structured regions, are stored in a 2x2 table. **c.** Tables showing results of statistical analysis of Odds Ratio with Fisher's test, using three disorder predictors. **d.** Plots of the Odds Ratio for human cell cycle-related non-CDK kinases and MAPK. **e.** Violin plots of the distribution of percentage of disordered residues per protein for CDK targets *vs* the rest of the phosphoproteome for human and yeast. Intrinsic disorder information of 13 different predictors was obtained from MobiDB, except for SPOT (calculated). Source data are provided as a Source Data file.

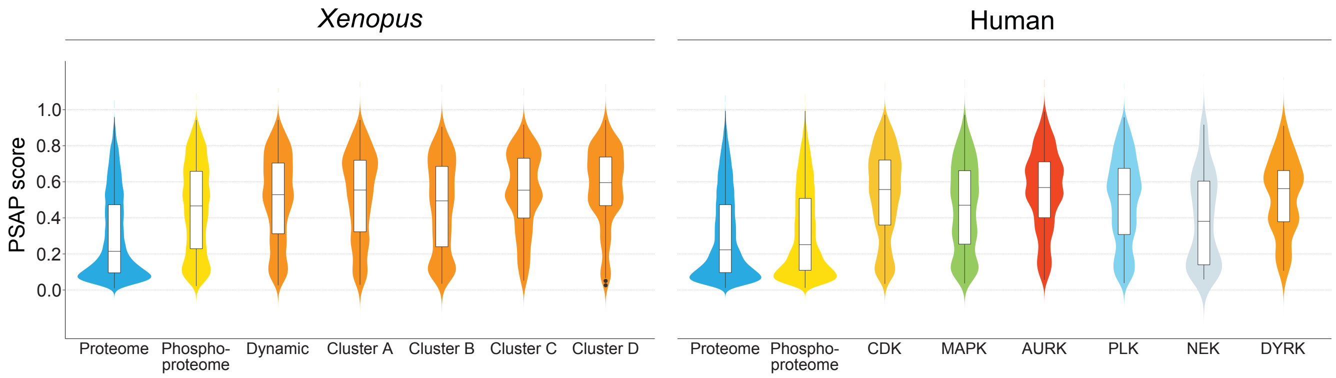

Supplementary Fig. 6. CDK-mediated phosphorylation regulates IDR phase separation propensity. Violin plots presenting PSAP score for *Xenopus* dynamic phosphoproteins (left), and human kinase targets, in comparison with total proteome and phosphoproteome. Boxplots center, median; lower and upper edges, 25% and 75% quartiles, respectively. Whiskers, data with the largest or smallest values not further than 1.5* interquartile range (IQR) from the upper or lower box limits, respectively. Beyond these values, data were not plotted, for clarity. No statistical test was performed to compare distributions of different sets.

a

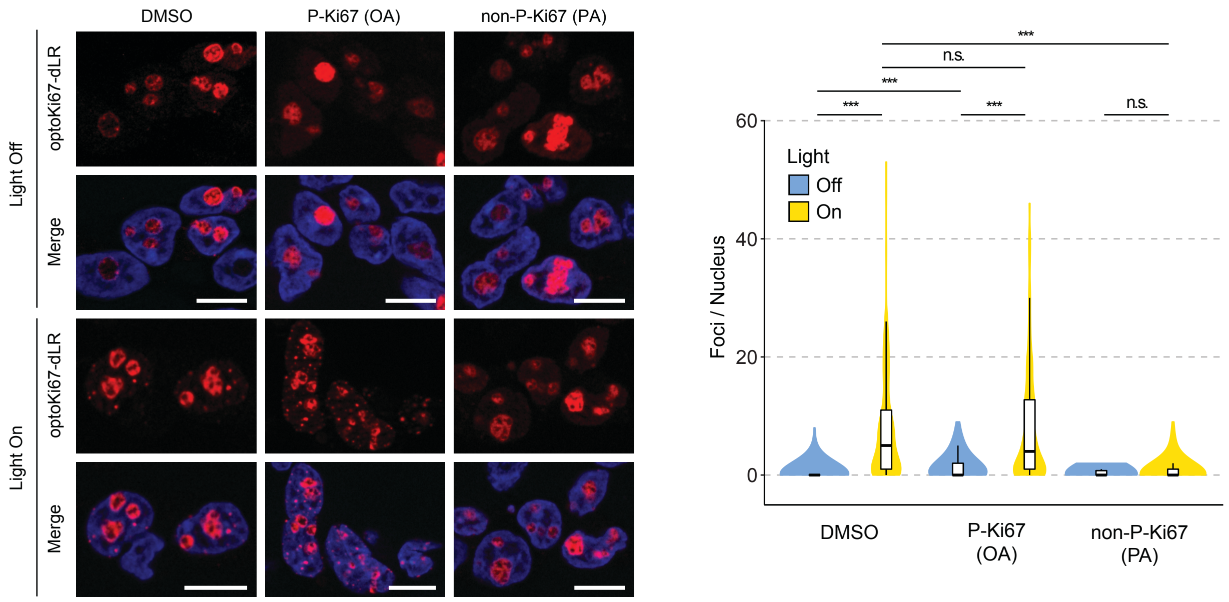

b

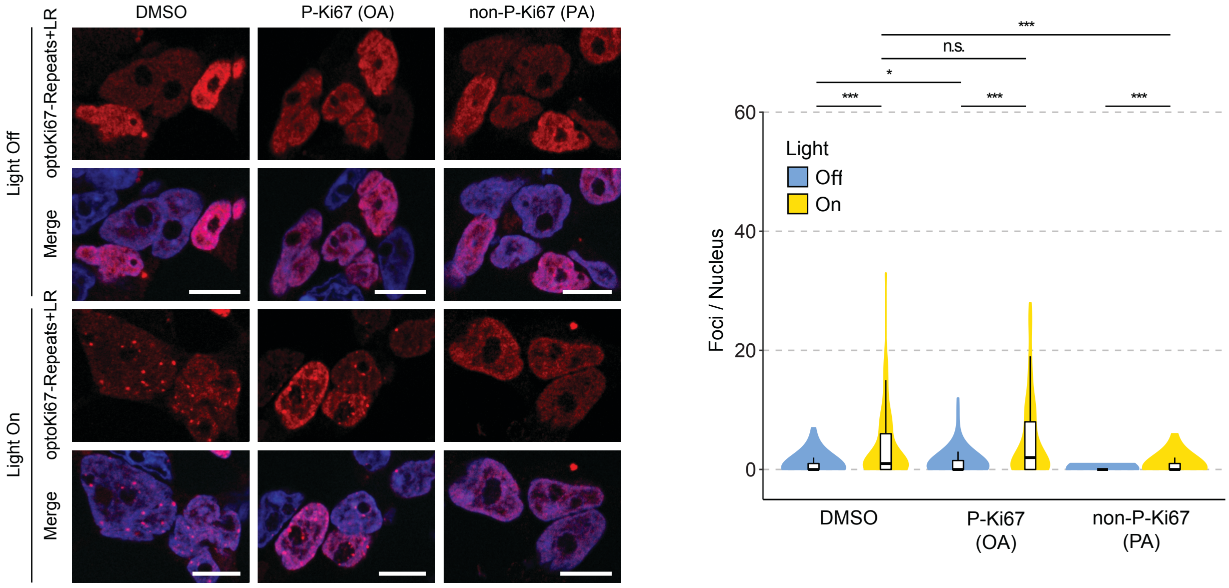

Supplementary Fig. 7. CDK-mediated phosphorylation regulates condensation of different Ki-67 subdomains. a, b. Left, representative fluorescent images of cells expressing opto-Ki-67 constructs with deleted LR domain, dLR, (**a**); or with deleted N-terminus, Repeats+LR (**b**), before (Light Off) and after (Light On) exposure to blue light. Cells were pretreated for 1h with either vehicle (DMSO), 0.5 μM okadaic acid (OA), to inhibit protein phosphatase 2A, or 5 μM purvalanol A (PA), to inhibit CDKs. DNA was stained with Hoechst 33258; scale bars, 10 μm; n=2 independent experiments. Right, quantification of results; the number of foci per nucleus was counted (>100 nuclei were analysed per condition). Statistical significance was assessed by one-way ANOVA on ranks (Kruskal–Wallis test) and pairwise *post-hoc* comparisons using the Mann–Whitney two-sided test. P-values were adjusted by the Benjamini-Hochberg method: a) DMSO Off vs DMSO On, p<2e-16; OA On vs DMSO On, p=0.814; OA On vs DMSO Off, p<2e-16; OA Off vs DMSO On, p=1.5e-10; OA Off vs DMSO Off, p=2.3e-05; OA Off vs, OA On, p=2.9e-09; PA On vs DMSO On, p<2e-16; PA On vs DMSO Off, p=0.392; PA On

vs OA On, p=8.9e-16; PA On vs OA Off, p=0.001; PA Off vs DMSO On, p<2e-16; PA Off vs DMSO Off, p=0.762; PA Off vs OA On, p<2e-16; PA Off vs OA Off, p=2.9e-05; PA Off vs PA On, p=0.584; b) DMSO Off vs DMSO On, p=8.0e-09; OA On vs DMSO On, p=0.032; OA On vs DMSO Off, p=4.4e-05; OA Off vs DMSO On, p=1.45e-09; OA Off vs OA On, p=3.3e-06; PA On vs DMSO On, p=3.3e-06; PA On vs DMSO Off, p<2e-16; PA On vs OA On, p=3.0e-11; PA On vs OA Off, p<2e-16; PA Off vs DMSO On, p=0.776; PA Off vs DMSO Off, p=1.4e-09; PA Off vs OA On, p=0.014; PA Off vs OA Off, p=2.8e-10; PA Off vs PA On, p=7.7e-06. Boxplots center, median; lower and upper edges, 25% and 75% quartiles, respectively. Whiskers, data with the largest or smallest values not further than 1.5* interquartile range (IQR) from the upper or lower box limits, respectively. Beyond these values, data were not plotted, for clarity, but were included in the statistical analysis. Source data are provided as a Source Data file.

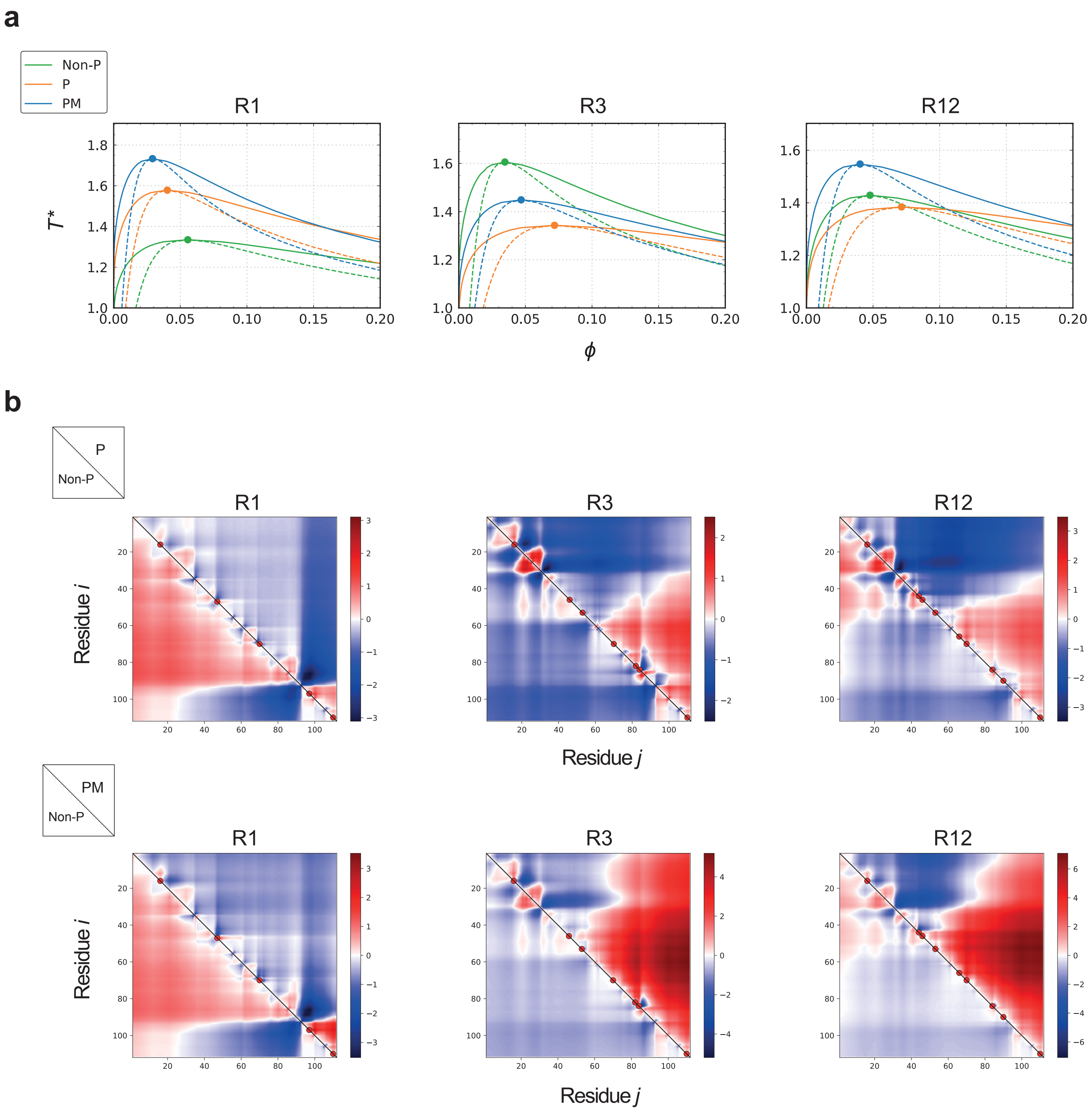

Supplementary Fig. 8. The sequence and degree of phosphorylation regulate phase separation propensity of Ki-67 repeats. a, b. Phase diagrams (**a**) and SCDM graphs (**b**) were generated for unmodified (Non-P, shown in green), phosphomimetic (phosphorylatable sites substituted by one negative charge; PM, shown in blue) and fully phosphorylated (phosphorylatable sites represented by two negative charges; P, shown in orange) sequences for three repeats R1, R3 and R12 of Ki-67 protein (sequence of repeats and phosphorylation sites as in [67]). These sequences were chosen as representative units to illustrate three different behaviours: phosphorylation (phosphomimetic or full) can enhance PS, as seen in R1, or reduce PS, as seen in R3; and phosphomimetic substitution can enhance PS, whereas full phosphorylation can reduce PS compared to the unmodified sequence, as seen in R12. SCDM graphs provide visual representation of single chain electrostatic interactions with unmodified sequences in the bottom triangle and phosphorylated sequence in the top triangle. Findings are consistent with PS tendencies for different units under different degrees of phosphorylation.

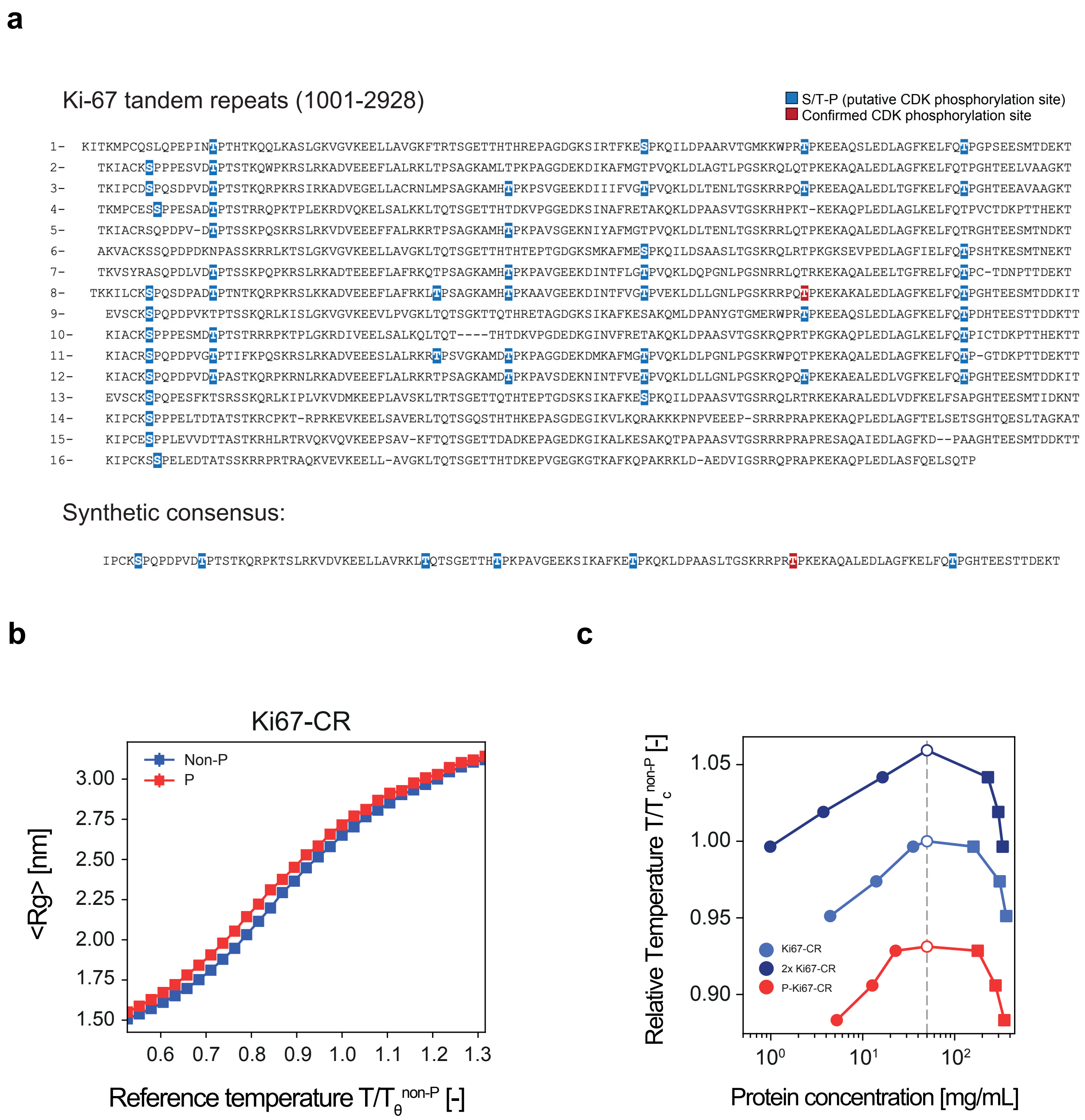

Supplementary Fig. 9. Phase behaviour of a synthetic Ki-67 consensus repeat protein. a. Alignment of human Ki-67 repeats, top, with the sequence of the consensus repeat depicted at the bottom. Confirmed and putative (Ser/Thr-Pro) CDK phosphorylation sites are highlighted in red and blue, respectively. **b.** Coarse grained single-chain MD simulations for the Ki-67 consensus repeat showing dependency of the radius of gyration (Rg) on the simulation temperature. **c.** Binodal curves from phase coexistence simulations of the Ki-67 consensus repeat sequence. For each temperature, filled circles indicate the dilute phase density and squares indicate the coexisting dense phase density. Empty circles indicate the fitted critical temperature (T_c) of each system. The T_c of the non-phosphorylated monomer (light blue empty circle) was the reference for the normalisation of the temperature values. The light grey dashed line indicates the total concentration used in the simulations. The reference temperature is the θ temperature of the non-phosphorylated molecule for full chain and consensus repeat, respectively. Error bars correspond to the standard error of the mean obtained with block analysis by subdividing the trajectory into 10 non-overlapping blocks. Source data are provided as a Source Data file.

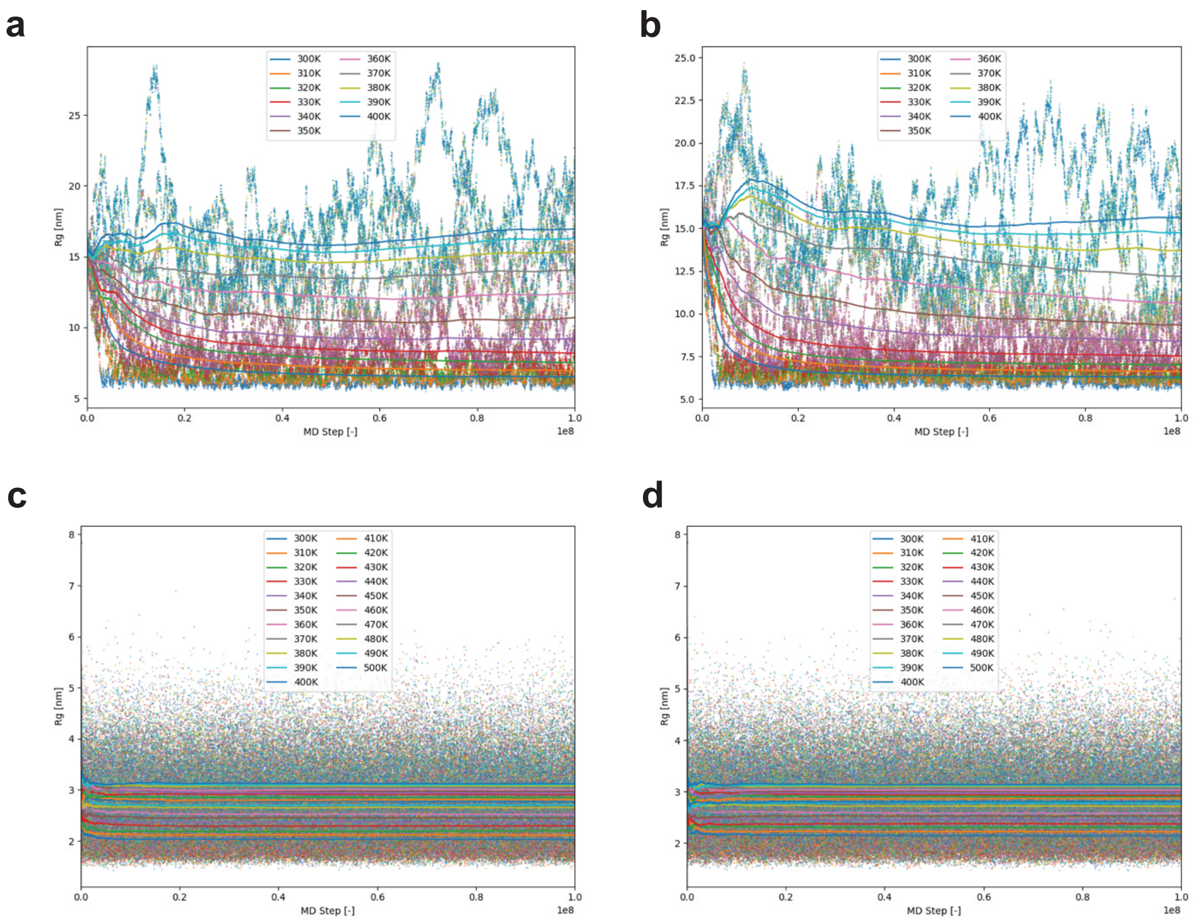

Supplementary Figure 10. Convergence analysis for single-chain MD simulations. Time series of the radius of gyration (Rg, dots) and convergence of the average value of Rg in time (lines) at constant temperature from PT-MD simulations of a single chain of (**a**) non-phosphorylated full-length Ki-67, (**b**) phosphorylated full-length Ki-67, (**c**) non-phosphorylated consensus repeat (CR) segment of Ki-67, and (**d**) phosphorylated CR segment of Ki-67. Source data are provided as a Source Data file.

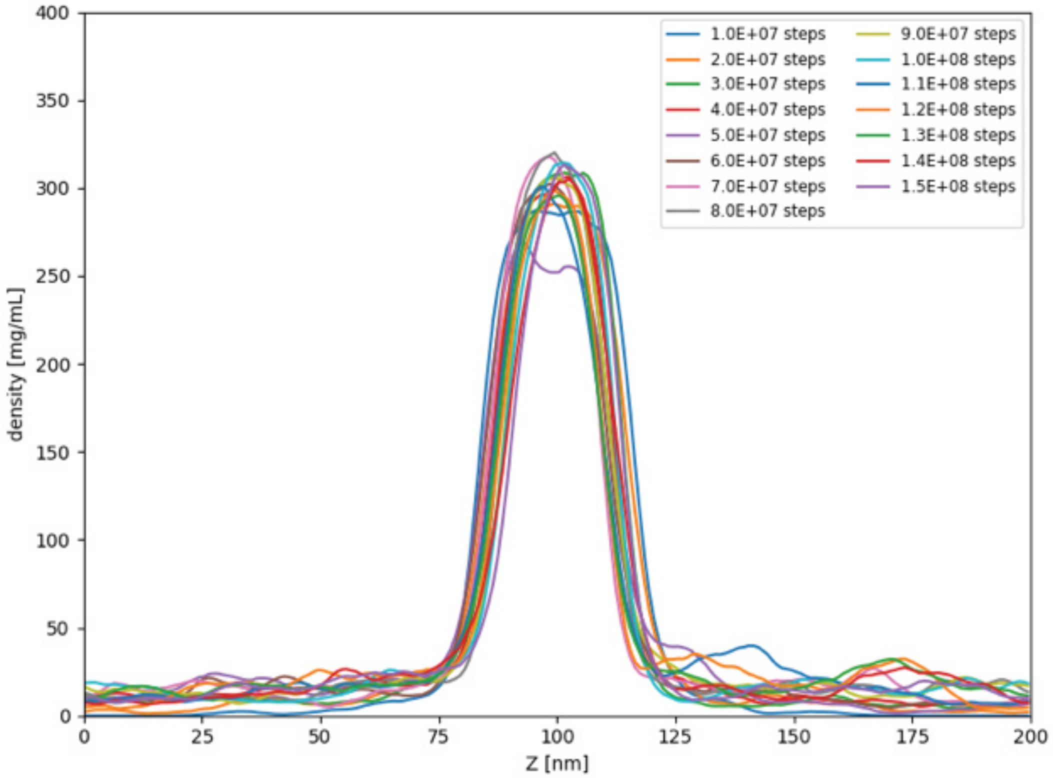

Supplementary Figure 11. Convergence of phase-coexistence simulations. Evolution of the density profile in time for the non-P monomer at relative temperature 0.97 [-], showing that the density profile of the system quickly equilibrates in the time scale of the simulations. Source data are provided as a Source Data file.
