# Supplementary material for: A cyclin-dependent kinase-mediated phosphorylation switch of disordered protein condensation
Source: Nat Commun. 2023 Oct 9;14:6316. doi: 10.1038/s41467-023-42049-0 (PMC10562473; doi:10.1038/s41467-023-42049-0)
Supplement: Supplementary file 3 — Description of Additional Supplementary Files [file 41467_2023_42049_MOESM3_ESM.pdf]

<u>Description of Additional Supplementary Files</u>

<u>Supplementary Data Legends</u>

Supplementary Data 1.

Phosphoproteomics data and analysis from single embryos during early *Xenopus laevis* embryonic cell cycles.

Supplementary Data 2.

High confidence sets of CDK substrates from human (manually curated) and *Saccharomyces cerevisiae.*

Supplementary Data 3.

Predictions of disordered residues across the entire proteomes of human, *Xenopus laevis* and *Saccharomyces cerevisiae.*

Supplementary Data 4.

A manually curated human membraneless organelle proteome.

Supplementary Data 5.

CDK phosphosite and structural data for selected membraneless organelle proteins shown in Fig. 5.

Supplementary Data 6.

Supplementary Files for Molecular Dynamics simulations. Folder names indicate the corresponding Figures in Main Text and Supplementary Information. For each system the initial configuration, final configuration, topology, index, and mdp files are given. Fig7a : Single chain simulations of full length Ki67; non-P : data to reproduce the simulations for non-phosphorylated full chain Ki67; P : data to reproduce the simulations for phosphorylated full chain Ki67. FigS9b : Single chain simulations of the consensus repeat segment of Ki67; non-P : data to reproduce the simulations for non-phosphorylated Ki67 consensus repeat segment; P : data to reproduce the simulations for phosphorylated Ki67 consensus repeat segment. FigS9c : Phase coexistence simulations of non-phosphorylated monomer, dimer and phosphorylated monomer of consensus repeat segment of Ki67; Ki67-CR : data to reproduce the simulations for the non-phosphorylated monomer of consensus repeat segment; 2x_Ki67-CR : data to reproduce the simulations for the non-phosphorylated dimer of consensus repeat segment; P-Ki67-CR : data to reproduce the simulations for the phosphorylated monomer of

consensus repeat segment. The tabulated potential for the electrostatic interactions with the Debye-Huckel screening (I=100mM) is provided in the table_nonBonded.xvg file.

Supplementary Movie Captions

Supplementary Movie 1.

Movie of Ki-67 FRAP experiment corresponding to the behaviour shown in Fig. 6c (top), with a cell in interphase expressing moderate levels of Ki-67.

Supplementary Movie 2.

Movie of Ki-67 FRAP experiment corresponding to the behaviour shown in Fig. 6c (middle) , with a cell in interphase expressing high levels of Ki-67.

Supplementary Movie 3.

Movie of Ki-67 FRAP experiment corresponding to the behaviour shown in Fig. 6c (bottom), with a cell in mitotic metaphase.

Supplementary Movie 4.

Representative trajectory from PT-MD simulation of full chain Ki-67 at relative temperature 1.1.

Supplementary Movie 5.

Phase coexistence MD simulation of non-phosphorylated monomers of the Ki-67 consensus repeat at a relative temperature of 0.98. This temperature is below the critical temperature (T_c) of the non-phosphorylated consensus repeat. Two phases are observed at equilibrium.

Supplementary Movie 6.

Phase coexistence MD simulation of phosphorylated monomers of the Ki-67 consensus repeat at a relative temperature of 0.98. This temperature is above the T_c of the phosphorylated repeat. A single homogeneous phase is observed at equilibrium.
