# Supplementary material for: A cyclin-dependent kinase-mediated phosphorylation switch of disordered protein condensation
Source: Nat Commun. 2023 Oct 9;14:6316. doi: 10.1038/s41467-023-42049-0 (PMC10562473; doi:10.1038/s41467-023-42049-0)
Supplement: Supplementary file 16 — Reporting Summary [file 41467_2023_42049_MOESM16_ESM.pdf]

nature portfolio

Corresponding author(s): Daniel Fisher, Maarten Altelaar

Last updated by author(s): Sep 6, 2023

Reporting Summary

Nature Portfolio wishes to improve the reproducibility of the work that we publish. This form provides structure for consistency and transparency in reporting. For further information on Nature Portfolio policies, see our Editorial Policies and the Editorial Policy Checklist.

Statistics

For all statistical analyses, confirm that the following items are present in the figure legend, table legend, main text, or Methods section.

n/a	Confirmed	
☐	☒	The exact sample size (*n*) for each experimental group/condition, given as a discrete number and unit of measurement
☐	☒	A statement on whether measurements were taken from distinct samples or whether the same sample was measured repeatedly
☐	☒	The statistical test(s) used AND whether they are one- or two-sided<br>*Only common tests should be described solely by name; describe more complex techniques in the Methods section.*
☐	☒	A description of all covariates tested
☐	☒	A description of any assumptions or corrections, such as tests of normality and adjustment for multiple comparisons
☐	☒	A full description of the statistical parameters including central tendency (e.g. means) or other basic estimates (e.g. regression coefficient) AND variation (e.g. standard deviation) or associated estimates of uncertainty (e.g. confidence intervals)
☐	☒	For null hypothesis testing, the test statistic (e.g. F, t, r) with confidence intervals, effect sizes, degrees of freedom and P value noted<br>*Give P values as exact values whenever suitable.*
☒	☐	For Bayesian analysis, information on the choice of priors and Markov chain Monte Carlo settings
☒	☐	For hierarchical and complex designs, identification of the appropriate level for tests and full reporting of outcomes
☒	☐	Estimates of effect sizes (e.g. Cohen's *d*, Pearson's *r*), indicating how they were calculated

Our web collection on statistics for biologists contains articles on many of the points above.

Software and code

Policy information about availability of computer code

Data collection	Data collection in all cases is described in the main text and/or in methods section. No software was employed for data retrieval.
Data analysis	Database search of the proteomic raw data was done using MaxQuant (v1.6.0.1). Further analysis was performed with TraDES v1, AmberTools v18, Perseus v1.6.0.2, Skyline v 21.1.0.146, Cytoscape v3.10.1 and the online STRING tool (v11.0).<br>All statistical analyses were performed with the R programming language (4.1.2 - GNU GPL v2) using the RStudio IDE (2021.09.2 Build 382 - GNU AGPL v3). Multiple libraries from "The Comprehensive R Archive Network" and "Bioconductor" repositories were used.<br>Coarse-grained molecular dynamics simulations were performed using GROMACS (2018.3).<br>NMR spectra were processed with TopSpin v3.5 (Bruker Biospin) and analysed using CCPN-Analysis software.<br>Inkscape (1.1.2 - GNU GPL v3) and Adobe Illustrator (Propietary software) were used for graphic edition.<br>Codes for rG-RPA calculation are uploaded in Github [https://github.com/MaxCalLab/IDPTheory/tree/main/rG-RPA]. Codes for phosphorylation and disorder analysis developed in this study are uploaded in Github [https://github.com/gero007/CDK-mediated-phosphorylation-of-IDRs].

For manuscripts utilizing custom algorithms or software that are central to the research but not yet described in published literature, software must be made available to editors and reviewers. We strongly encourage code deposition in a community repository (e.g. GitHub). See the Nature Portfolio guidelines for submitting code & software for further information.

Data

Policy information about availability of data

All manuscripts must include a data availability statement. This statement should provide the following information, where applicable:

- Accession codes, unique identifiers, or web links for publicly available datasets
- A description of any restrictions on data availability
- For clinical datasets or third party data, please ensure that the statement adheres to our policy

The mass spectrometry shotgun proteomics data generated in this study have been deposited in the ProteomeXchange Consortium via the PRIDE partner repository with the dataset identifier PXD023310 [https://proteomecentral.proteomexchange.org/cgi/GetDataset?ID=PXD023310]. The targeted proteomics data generated in this study have been deposited via Panorama with the identifier PXD026088 [https://proteomecentral.proteomexchange.org/cgi/GetDataset?ID=PXD026088]. The following public databases were used in this study: UniProt [https://www.uniprot.org/]; AlphaFold [https://alphafold.ebi.ac.uk/]; PhosphositePlus [https://www.phosphosite.org/] and RCSB Protein Data Bank PDB [https://www.rcsb.org]. Source data are provided with this paper.

Field-specific reporting

Please select the one below that is the best fit for your research. If you are not sure, read the appropriate sections before making your selection.

☒ Life sciences ☐ Behavioural & social sciences ☐ Ecological, evolutionary & environmental sciences

For a reference copy of the document with all sections, see nature.com/documents/nr-reporting-summary-flat.pdf

Life sciences study design

All studies must disclose on these points even when the disclosure is negative.

Sample size	No sample size estimations were used
Data exclusions	During the time-course of single-embryo phosphoproteomics, timepoints number 19,20 and 21 were excluded due to the impossibility of recording cell cycle phases (over-crowding).
Replication	Numbers of replicate experiments are stated in figure legends.
Randomization	No animals or patients that would require randomisation were involved.
Blinding	Blinding was not possible in this study as samples needed to be analysed with knowledge of the sample indentity (Westen blot, in vitro assays, IFs, cell transfection with different Ki-67 constructs).

Reporting for specific materials, systems and methods

We require information from authors about some types of materials, experimental systems and methods used in many studies. Here, indicate whether each material, system or method listed is relevant to your study. If you are not sure if a list item applies to your research, read the appropriate section before selecting a response.

Materials & experimental systems

n/a	Involved in the study	
☐	☒	Antibodies
☐	☒	Eukaryotic cell lines
☒	☐	Palaeontology and archaeology
☐	☒	Animals and other organisms
☒	☐	Human research participants
☒	☐	Clinical data
☒	☐	Dual use research of concern

Methods

n/a	Involved in the study	
☒	☐	ChIP-seq
☒	☐	Flow cytometry
☒	☐	MRI-based neuroimaging

Antibodies

Antibodies used

The following commercial antibodies were used in this study:
MeCP2 (Abcam, ab253197; IF, 1:500)
H3K9me3 (Abcam, ab8868; IF, 1:500)
Nucleolin (Abcam, ab22758; IF, 1:1000)
Nucleophosmin (Abcam, ab183340; IF, 1:1000)
GFP (Chromotek, PABG1; Western blot, 1:10 000)
Alexa Fluor 568 conjugated goat anti-rabbit (Invitrogen, A11011; IF, 1:1000)

	Goat anti-rabbit IgG (H+L) HRP (Thermo Fisher, # 32260; WB, 1:10 000)
Validation	All commercial antibodies used in this study are available and extensively validated by the company and others, and validation data are available on these company's website. None of these antibodies were validated by us, but we used them for the same species and applications as validated by the manufacturer.

Eukaryotic cell lines

Policy information about cell lines

Cell line source(s)	Flp-In™ T-REx™ 293 Cell Line - Invitrogen. HeLa cell line was obtained from A. Castro (CRBM, Montpellier, France), as described in Vera et al, Elife. 2015 Nov 27;4:e10115. doi: 10.7554/eLife.10115.
Authentication	The cell line is authenticated by the seller that states: "the 293 parental cell line was obtained from the American Type Culture Collection (ATCC®)". No cell line was authenticated by us.
Mycoplasma contamination	The cell lines were routinely monitored for mycoplasma contamination by the MycoAlertTM Mycoplasma Detection Kit. The cell lines were negative for mycoplasma
Commonly misidentified lines (See ICLAC register)	No commonly misidentified cell lines were used in this study.

Animals and other organisms

Policy information about studies involving animals; ARRIVE guidelines recommended for reporting animal research

Laboratory animals	For in vitro fertilisation experiments, eggs from adult X. laevis female frogs (aged >2 years; Nasco, catalog no. LM00535) were used, and demembranated sperm chromatin was prepared from the testes of adult X. laevis male frogs (purchased from the European Xenopus Resource Centre).<br>For egg extract preparation, adult Xenopus females were obtained from the Centre de Ressources Biologiques Xénopes (CRB) (Rennes, France), and housed in a dedicated aquatic facility located at the CRBM (Approval number B34-172-39)
Wild animals	No wild animals were used in this study.
Field-collected samples	No field-collected samples were used in this study.
Ethics oversight	For in vitro fertilisation experiments, all animal procedures and experiments were performed in accordance with national animal welfare laws and were reviewed by the Animal Ethics Committee of the Royal Netherlands Academy of Arts and Sciences (KNAW). All animal experiments were conducted under a project license granted by the Central Committee Animal Experimentation (CCD) of the Dutch government and approved by the Hubrecht Institute Animal Welfare Body (IvD), with project license number AVD80100201711044.<br>For egg extract preparation, animals were used following regulations according to the Direction Générale de la Recherche et Innovation and the French Ministry of Higher Education, Research and Innovation. All procedures were validated by the animal welfare committee of the Occitanie region.

Note that full information on the approval of the study protocol must also be provided in the manuscript.
